# Family psychosocial characteristics influencing criminal behaviour and mortality - possible mediating factors: a longitudinal study of male and female subjects in the Stockholm Birth Cohort

**DOI:** 10.1186/1471-2458-11-756

**Published:** 2011-10-02

**Authors:** Britt af Klinteberg, Ylva Almquist, Ulla Beijer, Per-Anders Rydelius

**Affiliations:** 1 Centre for Health Equity Studies, Stockholm University/Karolinska Institutet, Sveavägen 160, 5th floor, SE-106 91 Stockholm, Sweden; 2 Department of Psychology, Stockholm University, Stockholm, Sweden; 3 Department of Women's and Children's Health, Karolinska Institutet, Stockholm, Sweden; 4 Department of Public Health, Karolinska Institutet, Stockholm, Sweden

## Abstract

**Background:**

Family psychosocial characteristics in childhood have been associated with children's development into criminal behaviour and mortality. This study explored these possible relationships and examined alcohol and/or drug use and mental problems as possible mediating factors, highlighting gender-specific patterns.

**Methods:**

Data from Swedish subjects born in 1953 (n = 14,294) from the Stockholm Birth Cohort study were examined. Several indicators of adverse family factors and individual problems were included in the present study. The information was derived from various data sources, covering different periods. Gender-specific associations with incidence of criminality (1966-1980) and mortality (1981-2009) were analysed using logistic regression. Furthermore, the population attributable fraction (PAF) was calculated for all variables in the fully adjusted models which were positively related to the outcome.

**Results:**

Overall incidence of criminality and mortality was (m/f 32.3/6.6) and (m/f 6.1/3.5), respectively. The results showed that all aspects of family psychosocial and individual problems studied were associated with criminality for both genders. Among males, individual problems seemed to partly mediate these relations, but the associations remained statistically significant. Interestingly, the PAF analysis revealed a reduction in criminality of 17.5% when individual problems with alcohol and/or drug use were considered. Among females, a significant impact of alcohol and/or drug use on the association between family psychosocial characteristics and subsequent criminality was obtained. Inclusion of father's occupational class only somewhat reduced the estimates for the genders. Concerning male mortality, father's alcohol abuse was significantly related to an increased risk. When individual criminality was accounted for, the association was substantially reduced but remained statistically significant. Among females, when adjusting for family psychosocial factors, only the association between parents' mental problems and females' mortality was significant. None of the individual problem variables managed to explain this association.

**Conclusions:**

Family psychosocial characteristics were associated with both subsequent criminal behaviour and mortality. These connections were partly explained by individual risk factors, especially by alcohol and/or drug use. The practical implications of the findings point to the importance of addressing the individual's alcohol and/or drug use in reducing criminal behaviour, which would also lower the mortality rates.

## Background

Family psychosocial characteristics such as criminality, alcohol abuse, and mental problems are important to understand later criminal behaviour and premature death [1-5]. Most studies have, however, focused on male populations leaving it unclear whether these findings are also applicable to females. Moreover, there is a paucity of knowledge from longitudinal studies about individual factors that could possibly mediate these associations. The purpose of the present study is to examine gender differences in the influences of a variety of family psychosocial characteristics on the child's subsequent criminal behaviour and mortality and to see whether these influences could be mediated through different individual factors in the child.

Previous research has indicated that children from alcoholic families are at serious risk for developing various forms of problems as adults [6]. Already in an early follow-up study, the children of alcoholic fathers became criminals more often than other children [7]. A register-based study of about 84,800 adolescents and young adults in Denmark, covering the age period of 15-27 years, reported that parental alcohol abuse may influence several long-term consequences, such as increased self-destructive behaviours including drug addiction, hospitalization due to violence, and mortality [8]. Another study demonstrated that maternal and paternal alcohol use behaviours were positively linked with children's alcohol use behaviours at 14 and 171/2 years of age, among both genders [2]. Furthermore, a longitudinal study of Swedish male conscripts reported that there is a clear association between fathers' consumption patterns and their own alcohol consumption as well as risk of early death [9].

Children of parents who abuse alcohol or other drugs are moreover found to have higher rates of emotional problems [10]. Even for young children parental alcohol abuse, especially among fathers, has shown to be associated with negative effects as evidenced in subsequent anxiety/depression symptoms influenced via greater levels of marital aggression [11]. A cross-sectional study on a large group of medical students confirmed that there is a strong relationship among parental alcoholism, adverse childhood experience, and subsequent personal alcohol abuse [12]. The results of a study on the impact of adverse childhood experiences on a wide variety of health behaviours and outcomes, showed that adults who grew up with both an alcohol-abusing mother and father had the highest likelihood of multiple forms of adverse childhood experiences, such as abuse, neglect, and other household dysfunctions as compared to those who had only one- or no alcohol-abusing parent [13].

Furthermore, in a 33-year longitudinal study of children of alcoholics, non-delinquent sons of alcoholic men (COA's) had more alcoholic relatives, more environmental stress, more emotional and medical problems, and poorer adjustments than their non-COA peers [14]. However, the COA's did not demonstrate poor results on intelligence tests, nor increased behaviour problems related to hyperactivity, otherwise found to be a strong predictor of both subsequent alcohol problems and violence [15]. However, even if genes have a very strong impact on the development of substance abuse, there is an important interaction between genes and environmental influences in this process [16,17], as recently differently evidenced in male and female subjects [18]. In a study of individuals with substance use disorders and their adult first-degree relatives, there was an eight-fold increased risk of illicit drug disorders among the relatives as compared to their control group counterparts [19].

Generally, males are more likely to be at higher risk for early developed alcoholism, assumed to be more connected to biological and genetic factors, and distinguished from later developed alcoholism [20,21]. Early developed alcoholism is more frequently associated with additional problems like drug abuse and criminal behaviour than later developed alcoholism [22,21]. There are some indications of the existence of an early developed alcoholism related group also among female subjects [23]. A possible explanation for differences in reported results/indications between males and females could be that the hereditary form of alcoholism phenotypically expresses itself differently in females, despite the fact that the underlying genetic vulnerability might be the same.

It has furthermore been reported that individuals with addiction disorders have a higher occurrence of psychiatric and personality disorders [24]. This risk is nevertheless not equally distributed between the genders. While antisocial personality disorder dominates as the comorbid psychiatric diagnosis among alcohol dependent men, anxiety and depression are the most commonly reported diagnoses for women [25,26]. Studies have also shown a higher degree of alcoholism in families with a high degree of depression among women. To sum up, there seem to be differences concerning psychiatric comorbidity among alcohol-dependent- women and men.

An association between alcohol use or abuse and criminal behaviour, especially violent criminality, has also been reported in other groups [27-31]. In adolescents, higher alcohol consumption and more problematic alcohol and drug use was found in those with violence and conduct problems [32,33]. Strong connections between family history of alcohol abuse and violent offending were evidenced in a study by Linnoila and collaborators [34]. Notably, the vast majority of the offenders had committed crimes under the influence of alcohol.

Abused and neglected children, especially boys/males, had a higher likelihood of criminality, especially violence, compared with a control group with no experience of having been abused [35,36]. This is an important aspect in light of recent results reporting that many children have had an experience of living with parents having mental problems. A national survey in Canada that estimated the number of children exposed to parental psychiatric disorders, found that about 570,000 children under the age of 12 years were living with parents that had substance use, mood, or anxiety disorders [37]. Children with parents having mental problems have been found to have a higher risk to develop substance abuse and mental illness. Several studies have stated that children of parents with depression or substance misuse are at a higher risk of developing the same condition as the parent [38-42].

There is less research related to gender differences on the influences of family psychosocial characteristics [8]. However, boys with alcoholic parents tend to have an increased risk for criminal behaviour [6], and girls with maternal alcohol abuse and depression are found to have more serious mental problems than boys in the same situation [43]. Among outpatient adolescents and their parents, psychiatric problems among parents were found in 66% of the cases. The most common mental problem among fathers reporting alcohol abuse was depression (1/3). Fathers with both depression and alcohol problems were more frequent among the outpatient adolescent boys (29%) than girls (5%) [43].

Furthermore, a review of the effects of parental imprisonment on child antisocial behaviour and mental health showed that children of prisoners had about twice the risk of antisocial behaviour and poor mental health outcomes as compared to children whose parents were not imprisoned [3]. With regard to mortality, parental social class may have an influence on early death. In a prospective longitudinal study of men, where the fathers had a working-class occupation, 5% had died by age 48, and early death was strongly linked with criminal behaviour already at age 8-10 years [1]. In a study of offspring of mothers with psychotic disorders, females had lower all-cause mortality and mortality from unnatural causes than male subjects [44].

There are relatively few studies on populations of at-risk children; the vast majority has focused on data from parents in treatment settings. One of the few studies that used population-based data, from the National Longitudinal Alcohol Epidemiological Survey, estimated that approximately one out of four American children under the age of 17 years were exposed to alcohol abuse or dependence in the family (lifetime) [45]. Thus, there is a need for using large longitudinal databases for researching the underlying links between family lifestyle/characteristics and the development of criminal behaviour and mortality.

### Aim and hypothesis of the present study

The overall aim of the present study was to examine the influences of family psychosocial characteristics on the child's subsequent criminal behaviour and mortality using longitudinal data from the Stockholm Birth Cohort study. Three explicit research questions were identified: first, how different family psychosocial characteristics in childhood, such as father's criminality and alcohol abuse, as well as parental mental problems, may influence children's development into criminal behaviour and mortality; second, whether these possible relationships could potentially be mediated by the individual's own alcohol and/or drug use and mental problems; and, third, whether there are differences in these possible patterns by gender.

## Methods

### Subjects

Data were obtained from the *Stockholm Birth Cohort study *(SBC), created in 2004-05 by a probability matching between two longitudinal data sets: the *Stockholm Metropolitan Study *(SMS) and *The Swedish Work and Mortality Database *(WMD). The SMS consists of all children born in 1953, who lived in the greater Stockholm area in 1963 (n = 15,117). It contains both survey data and routine registry data from 1953 through 1985. The WMD is an anonymous database that includes registry-based information on work, income, labour market position and various health outcomes. It includes all persons born before 31 December 1985, who were alive and resident in Sweden on 31 December 1980 and/or 31 December 1990. The SMS data were de-identified in 1986. Through a probability matching procedure, based on a matching algorithm that included 13 variables identical to both datasets (containing information about e.g. occupational and housing conditions of the parents, as well as month of birth and gender of the cohort member), 14,294 individuals were positively matched and thus included in the SBC [for a more detailed description, see 46]. Ethical permission was obtained from the Stockholm Regional Ethics Committee (No 739-03-629).

Several different indicators of family psychosocial characteristics and individual problems were included in the present study. Therefore additional data were taken from various sources, such as the Stockholm City *Social Register*, covering the years 1953-1972. This registry contains information on parents' alcohol abuse and mental health problems, divided into three periods: 1953-1959, 1960-1965, and 1966-1972. In addition to this, it also contains information about the causes for decisions made by the Child Welfare Committee regarding the cohort members (e.g. alcohol and drug use and mental problems), registered on a yearly basis between 1962 and 1972. While this information reflects a rather high degree of severity of individual problems, it should be noted that the investigations conducted by the Committee could have various implications, ranging from *ad acta *(no measures taken) to social care. Since keeping a Social Register is mandatory in all Swedish municipalities, the coverage is good. However, there are some certain conditions that should be noted. Firstly, although all cohort members were born in 1953, some lived outside of the Metropolitan area before 1963 (approximately 18% of the original cohort). Moreover, a small proportion of families moved out of (and sometimes back into) the Metropolitan area after 1963. Any events during that time therefore were recorded in another municipality's register. While this hypothetically could cause an underestimation of the occurrence of family-related problems, sensitivity analysis did not demonstrate any significant differences in the main associations when excluding these individuals from the study population (data not presented). Secondly, all cases in the Social Register were filed under the head of household, but as soon as the cohort members reached the age of 16 they were considered their own head of household and thus had dossiers of their own (until they got married, whereby girls would have their cases filed under the name of their husband). Because the Social Register is only available up until 1972, this issue of marriage only involves a few cases. Thirdly, when a cohort member's family or the cohort member her-/himself had been registered in the Social Register for any reason, they may have had a higher probability of being registered for additional reasons [47]. Thus, this may cause a stronger correlation between the different types of information regarding problematic individual behaviour as well as adverse family psychosocial characteristics.

Another central data source used was the *National Crime Register *(PBR), containing data on convictions. Information on fathers' crimes is available for three time periods: pre-1953, 1953-1959, and 1959-1972 (first six months of 1972), and includes conditional sentences (probation), unconditional sentences (imprisonment), and exemption from punishment due to institutional psychiatric care or alcohol treatment. Yearly information on the cohort members' own crimes is available for the period between 1966 and 1984. Records for children under the age of 15 were, however, only filed by the police in extremely serious cases. Moreover, minor offences committed by individuals under the age of 18 were eliminated five years after registration, and serious offences 10 years later, if no further crimes were recorded. Crime data were divided into seven categories: violent crimes, stealing, fraud, vandalism, traffic crimes, narcotic crimes, and other crimes.

### Measures

The different variables concerning family psychosocial characteristics and individual problems are presented below. For the distribution of all variables according to gender, see Table 1.

**Table 1:** Distribution of family psychosocial characteristics, individuals problems and mortality according to gender (n = 14,294).

	Males (n = 7,305)		Females (n = 6,989)	
	
	n	%	n	%
*Family psychosocial characteristics*				
Father's criminality (1953-1959)				
No	7,058	96.6	6,732	96.3
Yes	247	3.4	257	3.7
Father's alcohol abuse (1953-1965)				
No	7,066	96.7	6,769	96.9
Yes	239	3.3	220	3.1
Parents' mental problems (1953-1965)				
No	6.944	95.1	6.697	95.8
Yes	361	4.9	292	4.2
Father's occupational class (1963)				
Upper and upper middle class	1,215	16.6	1,184	16.9
Middle class	3,060	41.9	3,002	43.0
Working class	2,810	38.5	2,644	37.8
Others	220	3.0	159	2.3
*Individual problems*				
Criminality - all crimes (1966-1980)				
No	4,943	67.7	6,530	93.4
Yes	2,362	32.3	459	6.6
Criminality - violent crimes (1966-1980)				
No	6,775	92.7	6,921	99.0
Yes	530	7.3	68	1.0
Criminality - non-violent crimes (1966-1980)				
No	5,014	68.6	6,548	93.7
Yes	2,291	31.4	441	6.3
Alcohol and/or drug use (1966-1972)				
No	6,973	95.5	6,807	97.4
Yes	332	4.5	182	2.6
Mental problems (1966-1972)				
No	7,149	97.9	6.897	98.7
Yes	156	2.1	92	1.3
*Mortality*				
Mortality (1981-2009)				
No	6,861	93.9	6,742	96.5
Yes	444	6.1	247	3.5

#### Family psychosocial characteristics

##### Father's criminality

A dichotomous variable, indicating if the father had committed any crime between 1953 and 1959 (child age 0-6), was created. Here, all types of sentence (i.e. conditional, unconditional, and exemption from punishment) were included.

##### Father's alcohol abuse

Information on the father's alcohol abuse between 1953 and 1965 (child age 0-13) was included (regardless of whether or not the father was subject to institutional treatment or action by the temperance committee), categorised into 'yes' or 'no'.

##### Parents' mental health problems

This information had already been categorised into 'psychiatric problems or depressed', 'receiving psychiatric treatment,' and 'committed suicide' (no additional information on the type of problem was thus available). Individuals whose father and/or mother were registered in any of these categories between 1953 and 1965 (child age 0-12) were considered as having a parent with mental health problems.

##### Father's occupational class

This fourth type of information concerning family circumstances was included primarily as a control variable: *father's occupational class *in 1963 (child age 10). The categories were 'upper' (upper and upper middle class), 'middle' (officials and entrepreneurs), 'lower' (skilled and unskilled workers) and 'others' (unemployed, students and pensioners).

#### Individual problems

##### Criminality

Two variables based on information about the individual's criminality were constructed. The first included all crimes that the cohort member committed between 1966 and 1980 (age 13-27), regardless of type. The second included only violent crimes (crimes against a person, e.g. physical violence and treats of physical violence such as assault, rape, robbery, molestation, and unlawful intrusion) during the same period.

##### Alcohol and/or drug use

This information indicates whether or not actions were taken by the Child Welfare Committee due to the individual's use of alcohol and/or drugs any time during the period 1966-1972 (age 13-19).

##### Mental problems

Information on the individual's mental problems, covering the period 1966-1972 (age 13-19), refers to any actions taken by the Child Welfare Committee due to such problems. No explicit information regarding the type or degree of severity is available, explaining why this category is very broad.

##### Mortality

Information about mortality was available through the *Cause of Death Register*. Since the probability matching required that cohort members had to be alive at the end of 1980, the information covers the period 1981 through 2009 (age 28-56). All deaths, regardless of diagnosis, are included. Causes of death in these (young) ages are most likely linked to behavioural and lifestyle-related disturbances (e.g. alcohol and drug abuse), as well as external causes (e.g. suicide or accidents). Due to the rarity of mortality at these ages (m/f 6.1/3.5) it was not possible to examine specific causes of death.

### Statistical analysis

The analyses were carried out using logistic regression, producing odds ratios. Since the association may be assumed to differ according to gender, all analyses were gender-specific. Table 2 (males) and Table 3 (females) show the association between adverse family psychosocial characteristics and criminality (all crimes). The first column demonstrates the 'crude' (i.e. unadjusted) association, whereas Model 1 takes into account all family psychosocial factors simultaneously. In Model 2, there is additional adjustment for father's occupational class. Model 3 takes into account the individual's own alcohol and drug use, and Model 4 incorporates the effect of the individual's own mental problems. In Model 5, all variables are adjusted for simultaneously. Table 4 (males) and Table 5 (females) demonstrate the associations between adverse family psychosocial characteristics and mortality, taking individual problems into account. The first column presents the crude association between all family psychosocial characteristics and individual factors, in relation to mortality. In Model 1, the three indicators of adverse family psychosocial characteristics are included simultaneously, whereas in Model 2 father's occupational class is also included. Models 3-6 additionally adjust for one individual factor at a time. In the subsequent columns, Model 7 and Model 8, all factors are included (*note: *all crimes and violent crimes are separated due to overlap).

**Table 2:** Family psychosocial characteristics and criminality (all crimes) among males, adjusting for individual problems.

	All crimes (1966-1980)						
	
	Crude^a^ (95% CI)	Model 1^b^ (95% CI)	Model 2^c^ (95% CI)	Model 3^d^ (95% CI)	Model 4^e^ (95% CI)	Model 5^f^ (95% CI)	PAF
*Family psychosocial characteristics*							
							
Father's criminality (1953-1959)							
No (ref.)	1.00	1.00	1.00	1.00	1.00	1.00	
Yes	2.10 (1.63-2.71)	1.70 (1.30-1.21)	1.53 (1.17-1.99)	1.41 (1.07-1.86)	1.54 (1.17-2.01)	1.42 (1.08-1.88)	-2.05%
							
Father's alcohol abuse (1953-1965)							
No (ref.)	1.00	1.00	1.00	1.00	1.00	1.00	
Yes	3.03 (2.33-3.93)	2.41 (1.83-3.16)	2.06 (1.56-2.71)	1.84 (1.38-2.47)	1.93 (1.45-2.56)	1.77 (1.32-2.37)	-2.96%
							
Parents' mental problems (1953-1965)							
No (ref.)	1.00	1.00	1.00	1.00	1.00	1.00	
Yes	2.12 (1.71-2.62)	1.77 (1.42-2.20)	1.56 (1.25-1.95)	1.48 (1.17-1.86)	1.43 (1.13-1.79)	1.40 (1.10-1.77)	-2.89%
							
Father's occupational class (1963)							
Upper and upper middle class (ref.)	1.00		1.00	1.00	1.00	1.00	
Middle class	1.58 (1.35-1.85)		1.53 (1.30-1.79)	1.47 (1.25-1.73)	1.52 (1.29-1.78)	1.47 (1.25-1.73)	
Working class	2.45 (2.09-2.87)		2.26 (1.93-2.65)	2.08 (1.76-2.45)	2.23 (1.90-2.62)	2.08 (1.77-2.45)	
Others	3.39 (2.51-4.57)		2.73 (2.01-3.71)	2.63 (1.92-3.60)	2.64 (7.28-18.2)	2.56 (1.87-3.52)	-1.85%
							
*Individual problems*							
							
Alcohol and/or drug use (1966-1972)							
No (ref.)	1.00			1.00		1.00	
Yes	22.3 (15.5-32.3)			19.5 (13.5-28.2)		15.2 (10.5-22.2)	-17.5%
							
Mental problems (1966-1972)							
No (ref.)	1.00				1.00	1.00	
Yes	13.5 (8.55-21.2)				11.5 (7.28-18.2)	4.66 (2.82-7.72)	-4.53%

Odds ratios from logistic regression analysis (n = 7,305).

^a ^No adjustment, ^b ^Mutual adjustment for family psychosocial characteristics, ^c ^Mutual adjustment for family psychosocial characteristics + father's occupational class, ^d ^Mutual adjustment for family psychosocial characteristics + father's occupational class + individual's alcohol and/or drug use, ^e ^Mutual adjustment for family psychosocial characteristics + father's occupational class + individual's mental problems, ^f ^Mutual adjustment for all variables

**Table 3:** Family psychosocial characteristics and criminality (all crimes) among females, adjusting for individual problems.

	All crimes (1966-1980)						
	
	Crude^a^ (95% CI)	Model 1^b^ (95% CI)	Model 2^c^ (95% CI)	Model 3^d^ (95% CI)	Model 4^e^ (95% CI)	Model 5^f^ (95% CI)	PAF
*Adverse family psychosocial characteristics*							
							
Father's criminality (1953-1959)							
No (ref.)	1.00	1.00	1.00	1.00	1.00	1.00	
Yes	2.52 (1.75-3.61)	1.83 (1.25-2.69)	1.71 (1.17-2.52)	1.31 (0.83-2.05)	1.68 (1.12-2.50)	1.32 (0.84-2.07)	-0.42%
							
Father's alcohol abuse (1953-1965)							
No (ref.)	1.00	1.00	1.00	1.00	1.00	1.00	
Yes	3.59 (2.53-5.10)	2.69 (1.84-3.93)	2.51 (1.72-3.67)	2.08 (1.34-3.21)	2.39 (1.61-3.54)	2.06 (1.32-3.20)	-0.83%
							
Parents' mental problems (1953-1965)							
No (ref.)	1.00	1.00	1.00	1.00	1.00	1.00	
Yes	2.45 (1.74-3.46)	1.84 (1.28-2.65)	1.70 (1.18-2.46)	1.43 (0.94-2.17)	1.49 (1.01-2.19)	1.40 (0.92-2.13)	-0.54%
							
Father's occupational class (1963)							
Upper and upper middle class (ref.)	1.00		1.00	1.00	1.00	1.00	
Middle class	0.97 (0.72-1.32)		0.92 (0.68-1.25)	0.79 (0.57-1.08)	0.90 (0.66-1.22)	0.80 (0.58-1.10)	
Working class	1.69 (1.27-2.26)		1.50 (1.11-2.01)	1.24 (0.91-1.69)	1.39 (1.03-1.87)	1.24 (0.91-1.68)	
Others	2.31 (1.33-4.02)		1.74 (0.99-3.07)	1.54 (0.83-2.84)	1.71 (0.96-3.05)	1.54 (0.83-2.85)	-0.33%
							
*Individual problems*							
							
Alcohol and/or drug use (1966-1972)							
No (ref.)	1.00			1.00		1.00	
Yes	33.1 (24.0-45.7)			29.2 (21.0-40.6)		22.9 (15.9-33.1)	-2.85%
							
Mental problems (1966-1972)							
No (ref.)	1.00				1.00	1.00	
Yes	18.9 (12.4-28.8)				15.6 (10.1-24.0)	2.30 (1.28-4.13)	-0.41%

Odds ratios from logistic regression analysis (n = 6,989).

^a ^No adjustment, ^b ^Mutual adjustment for family psychosocial characteristics, ^c ^Mutual adjustment for family psychosocial characteristics + father's occupational class, ^d ^Mutual adjustment for family psychosocial characteristics + father's occupational class + individual's alcohol and/or drug use, ^e ^Mutual adjustment for family psychosocial characteristics + father's occupational class + individual's mental problems, ^f ^Mutual adjustment for all variables

**Table 4:** Adverse family psychosocial characteristics, individual problems, and mortality among males.

	Mortality (1981-2009)									
	
	Crude^a^ (95% CI)	Model 1^b^ (95% CI)	Model 2^c^ (95% CI)	Model 3^d^ (95% CI)	Model 4^e^ (95% CI)	Model 5^f^ (95% CI)	Model 6^g^ (95% CI)	Model 7^h^ (95% CI)	Model 8^i^ (95% CI)	PAF
*Family psychosocial characteristics*										
Father's criminality (1953-1959)										
No (ref.)	1.00	1.00	1.00	1.00	1.00	1.00	1.00	1.00	1.00	
Yes	1.46 (0.92-2.30)	1.10 (0.68-1.78)	1.05 (0.65-1.69)	0.97 (0.60-1.57)	0.97 (0.59-1.57)	0.90 (0.55-1.48)	1.03 (0.64-1.68)	0.88 (0.54-1.44)	0.89 (0.54-1.46)	
Father's alcohol abuse (1953-1965)										
No (ref.)	1.00	1.00	1.00	1.00	1.00	1.00	1.00	1.00	1.00	
Yes	3.00 (2.08-4.31)	2.87 (1.94-4.24)	2.53 (1.70-3.76)	2.16 (1.45-3.22)	2.05 (1.36-3.07)	2.11 (1.40-3.19)	2.31 (1.54-3.46)	1.92 (1.27-2.90)	1.88 (1.24-2.86)	-0.69%
Parents' mental problems (1953-1965)										
No (ref.)	1.00	1.00	1.00	1.00	1.00	1.00	1.00	1.00	1.00	
Yes	1.43 (0.97-2.10)	1.10 (0.73-1.66)	1.02 (0.68-1.54)	0.93 (0.62-1.40)	0.95 (0.62-1.43)	0.93 (0.61-1.41)	0.89 (0.58-1.35)	0.86 (0.56-1.30)	0.88 (0.58-1.35)	
Father's occupational class (1963)										
Upper and upper middle class (ref.)	1.00		1.00	1.00	1.00	1.00	1.00	1.00	1.00	
Middle class	1.35 (0.98-1.86)		1.33 (0.97-1.84)	1.21 (0.88-1.68)	1.27 (0.92-1.75)	1.25 (0.91-1.73)	1.31 (0.95-1.81)	1.18 (0.85-1.63)	1.23 (0.89-1.70)	
Working class	1.72 (1.26-2.36)		1.62 (1.18-2.22)	1.34 (0.97-1.85)	1.44 (1.04-1.99)	1.38 (1.00-1.90)	1.56 (1.14-2.15)	1.23 (0.89-1.71)	1.32 (0.95-1.82)	
Others	2.93 (1.77-4.83)		1.39 (1.42-4.00)	1.93 (1.14-3.25)	2.09 (1.24-3.54)	2.24 (1.32-3.79)	2.30 (1.37-3.87)	1.96 (0.16-3.31)	2.09 (1.23-3.55)	-0.50%
*Individual problems*										
All crimes (1966-1980)										
No (ref.)	1.00			1.00				1.00		
Yes	2.96 (2.43-3.59)			2.75 (2.26-3.36)				2.12 (1.71-2.62)		-8.49%
Violent crimes (1966-1980)										
No (ref.)	1.00				1.00				1.00	
Yes	3.71 (2.89-4.77)				3.24 (2.50-4.20)				2.00 (1.49-2.67)	-1.80%
Alcohol and/or drug use (1966-1972)										
No (ref.)	1.00					1.00		1.00	1.00	
Yes	6.83 (5.24-8.90)					6.21 (4.72-8.15)		3.94 (2.88-5.39)	4.43 (3.22-6.11)	-2.03%
Mental problems (1966-1972)										
No (ref.)	1.00						1.00	1.00	1.00	
Yes	4.77 (3.23-7.03)						4.10 (2.74-6.12)	1.34 (0.85-2.10)	1.36 (0.86-2.16)	-0.19%

Odds ratios from logistic regression analysis (n = 7,305).

^a ^No adjustment, ^b ^Mutual adjustment for family psychosocial characteristics, ^c ^Mutual adjustment for family psychosocial characteristics + father's occupational class, ^d ^Mutual adjustment for family psychosocial characteristics + father's occupational class + all crimes, ^e ^Mutual adjustment for family psychosocial characteristics + father's occupational class + violent crimes

^f ^Mutual adjustment for family psychosocial characteristics + father's occupational class + alcohol and/or drug use, ^g ^Mutual adjustment for family psychosocial characteristics + father's occupational class + mental problems, ^h ^Mutual adjustment for family psychosocial characteristics + father's occupational class + all crimes + alcohol and/or drug use + mental problems

^i ^Mutual adjustment for family psychosocial characteristics + father's occupational class + violent crimes + alcohol and/or drug use + mental problems

**Table 5:** Adverse family psychosocial characteristics, individual problems, and mortality among females.

	Mortality (1981-2009)									
	
	Crude^a^ (95% CI)	Model 1^b^ (95% CI)	Model 2^c^ (95% CI)	Model 3^d^ (95% CI)	Model 4^e^ (95% CI)	Model 5^f^ (95% CI)	Model 6^g^ (95% CI)	Model 7^h^ (95% CI)	Model 8^i^ (95% CI)	PAF
*Family psychosocial characteristics*										
Father's criminality (1953-1959)										
No (ref.)	1.00	1.00	1.00	1.00	1.00	1.00	1.00	1.00	1.00	
Yes	1.87 (1.11-3.15)	1.45 (0.84-2.52)	1.41 (0.81-2.45)	1.31 (0.75-2.27)	1.36 (0.78-2.36)	1.16 (0.65-2.06)	1.38 (0.79-2.40)	1.17 (0.66-2.07)	1.16 (0.65-2.07)	-0.16%
Father's alcohol abuse (1953-1965)										
No (ref.)	1.00	1.00	1.00	1.00	1.00	1.00	1.00	1.00	1.00	
Yes	2.22 (1.31-3.75)	1.57 (0.89-2.77)	1.53 (0.87-2.69)	1.26 (0.71-2.24)	1.50 (0.84-2.65)	1.26 (0.70-2.26)	1.46 (0.83-2.57)	1.14 (0.63-2.06)	1.27 (0.71-2.29)	-0.15%
Parents' mental problems (1953-1965)										
No (ref.)	1.00	1.00	1.00	1.00	1.00	1.00	1.00	1.00	1.00	
Yes	2.86 (1.87-4.38)	2.51 (1.61-3.92)	2.40 (1.53-3.76)	2.21 (1.40-3.49)	2.31 (1.47-3.65)	2.15 (1.36-3.40)	2.19 (1.39-3.45)	2.14 (1.34-3.37)	2.13 (1.35-3.40)	-0.93%
Father's occupational class (1963)										
Upper and upper middle class (ref.)	1.00		1.00	1.00	1.00	1.00	1.00	1.00	1.00	
Middle class	0.86 (0.59-1.25)		0.82 (0.56-1.20)	0.83 (0.57-1.21)	0.82 (0.56-1.20)	0.78 (0.53-1.13)	0.82 (0.56-1.19)	0.79 (0.54-1.15)	0.77 (0.53-1.13)	
Working class	1.16 (0.81-1.68)		1.05 (0.72-1.52)	0.99 (0.68-1.45)	1.03 (0.71-1.50)	0.94 (0.65-1.38)	0.99 (0.68-1.44)	0.92 (0.63-1.35)	0.94 (0.64-1.37)	
Others	1.87 (0.92-3.81)		1.43 (0.69-2.97)	1.32 (0.63-2.76)	1.39 (0.66-2.90)	1.34 (0.64-2.81)	1.41 (0.68-2.93)	1.30 (0.62-2.73)	1.34 (0.64-2.80)	-0.26%
*Individual problems*										
All crimes (1966-1980)										
No (ref.)	1.00			1.00				1.00		
Yes	4.15 (3.01-5.73)			3.74 (2.69-5.20)				2.25 (1.51-3.35)		-1.57%
Violent crimes (1966-1980)										
No (ref.)	1.00				1.00				1.00	
Yes	8.91 (5.01-15.8)				8.03 (4.47-14.4)				2.85 (1.44-5.65)	-0.30%
Alcohol and/or drug use (1966-1972)										
No (ref.)	1.00					1.00		1.00	1.00	
Yes	8.02 (5.45-11.8)					7.07 (4.74-10.6)		3.70 (2.17-6.31)	4.63 (2.77-7.75)	-0.92%
Mental problems (1966-1972)										
No (ref.)	1.00						1.00	1.00	1.00	
Yes	7.08 (4.16-12.1)						5.88 (3.41-10.1)	1.59 (0.82-3.10)	1.61 (0.82-3.17)	-0.17%

Odds ratios from logistic regression analysis (n = 6,989).

^a ^No adjustment, ^b ^Mutual adjustment for family psychosocial characteristics, ^c ^Mutual adjustment for family psychosocial characteristics + father's occupational class, ^d ^Mutual adjustment for family psychosocial characteristics + father's occupational class + all crimes, ^e ^Mutual adjustment for family psychosocial characteristics + father's occupational class + violent crimes

^f ^Mutual adjustment for family psychosocial characteristics + father's occupational class + alcohol and/or drug use, ^g ^Mutual adjustment for family psychosocial characteristics + father's occupational class + mental problems, ^h ^Mutual adjustment for family psychosocial characteristics + father's occupational class + all crimes + alcohol and/or drug use + mental problems

^i ^Mutual adjustment for family psychosocial characteristics + father's occupational class + violent crimes + alcohol and/or drug use + mental problems

In addition, the population attributable fraction (PAF) was calculated for all terms in the fully adjusted models which were positively related to the outcome. Population attributable fraction refers to the proportion of the studied outcome that may be ascribed a given exposure (if the exposure would be causal). The PAF:s were derived by using the aflogit command in Stata 11.0, which is designed for logistic regression models [48]. The percentages are added to the final column of Table 2, 3, 4 and 5 (note: the PAF for 'all crimes' is based on Model 7 and the PAF for 'violent crimes' is based on Model 8, while the remaining PAF:s are based on a model that includes both these variables). The PAF results given are non-overlapping.

## Results

### Associations between family psychosocial characteristics and criminality

As the first column in Table 2 demonstrates, all aspects of family psychosocial characteristics and individual problems are associated with criminality among *males *at a statistically significant level. For example, it is more than twice as common to be convicted for a crime among males whose father was a criminal (OR 2.10) or whose parents had mental problems (OR 2.12). Moreover, among those whose father abused alcohol, it is three times as common to subsequently be convicted. In the next column (Model 1), the three indicators of adverse family psychosocial characteristics have been included in the same model. The results show that they are independently associated with criminality among males. The inclusion of father's occupational class (Model 2) leads to some reduction of the estimates. In the next column (Model 3), the individual's alcohol and/or drug use is added to the model. This causes an additional decrease of the estimates for the aspects of adverse family psychosocial characteristics, but the main findings remain intact. When information on the individual's mental problems is included instead (Model 4), the estimates are largely unchanged, indicating a paucity of mediating effects on the associations obtained. In the following column (Model 5), all variables are included simultaneously. While the estimates are further reduced, all aspects of family psychosocial characteristics remain significantly associated with the individual's subsequent criminality.

In Table 3 the corresponding associations for *females *are demonstrated. Here as well, all aspects of family psychosocial characteristics and individuals problems are found to be associated with females' criminality. The second column (Model 1) includes the three indicators of adverse family psychosocial characteristics, which lead to a substantial decrease of the estimates. The estimates are further reduced when adjusted for father's occupational class (Model 2). In the fourth column (Model 3), the individual's alcohol and/or drug use is added to the model. This brings the association between father's criminality and females' own criminality, on the one hand, and parents' mental problems and females' own criminality, on the other hand, to a non-significant level. This reduction is not as great in the next column (Model 4) when there is an adjustment for individual mental problems. In the next column (Model 5), when all variables are included simultaneously, the decrease of the estimates primarily reflects the impact of females' own alcohol and/or drug use on the association between adverse family psychosocial characteristics and females' own subsequent criminality.

### Associations between family psychosocial characteristics and mortality

Table 4 concerns the association between adverse family psychosocial characteristics and subsequent mortality among *males*. The first column demonstrates that of the three indicators of family psychosocial characteristics, only father's alcohol abuse is significantly related to mortality. It is three times as common (OR 3.00) among males with fathers who abused alcohol to die sometime between age 28 and age 56 as compared to other males. When all three indicators are adjusted for simultaneously (Model 1), the estimates decrease to some extent. They are further reduced when father's occupational class is added in the next column (Model 2). In the next column (Model 3), the individual's own criminality is accounted for: the association between father's alcohol abuse and mortality is substantially reduced but remains statistically significant. A slightly higher decrease is observed in the next column (Model 4), when only violent crimes are concerned. The following column (Model 5) adjusts for alcohol and/or drug use, which also leads to a decrease of the strength in the association. Where the individual's mental problems are considered, however, the estimate is not lowered as much (Model 6). In the next two columns, all variables are included simultaneously. However, the association between father's alcohol abuse and mortality still remains highly statistically significant.

The corresponding findings for *females *are shown in Table 5. As the first column demonstrates, all factors except father's occupational class are associated with subsequent mortality. The second column (Model 1) includes the three indicators of adverse family psychosocial characteristics simultaneously, this leaves the estimates for father's criminality and father's alcohol abuse at a statistically non-significant level. The association between parents' mental problems and females' mortality remains strong and statistically significant. In the next column (Model 2), father's occupational class is adjusted for, resulting in some reduction of the estimates. The following four columns adjust for different individual factors, separately: all crimes (Model 3), violent crimes (Model 4), alcohol and drug use (Model 5) and mental problems (Model 6). None of these explanatory variables manage to explain the association between parents' mental problems and females' subsequent mortality, although the estimate decreases to some extent. The next two columns include all variables. In both cases, it is still more than twice as common among females whose parents had mental problems to die between age 28 and age 56.

### Population attributable fractions

The results from the last column of Table 2 demonstrate the population attributable fractions for the different factors. For example, 2% of the cases of criminality among males could be avoided if the effect of father's alcohol abuse was eliminated. The corresponding PAF for the individual's alcohol and drug use is 17.5% (or 413 persons). The similar pattern is demonstrated for females (Table 3), although the point estimates for the PAF:s are much lower. Worth noticing is that in addition to the PAF:s being lower, the crime rate among females is also considerably lower, explaining why eliminating the exposure of certain factors would affect even fewer persons (e.g., 13 persons if the exposure to alcohol and drug use was removed).

In Table 4 the last column shows the population attributable fractions for mortality among males. The results demonstrate that only 0.7% of the mortality cases among men may be attributed to father's alcohol abuse. Of the factors related to individual problems, 8.5% of the cases in mortality (or 37 persons) are attributable to the individual's own criminality (all crimes) whereas the corresponding figure for alcohol and drug use is 2.0%. Concerning females' mortality (Table 5), the population attributable fractions for family psychosocial characteristics factors are very small. The pattern shown for males' individual problems emerges in females as well. However, again, the estimates are considerably lower: approximately 1.6% and 0.9% of the deaths among females may be attributed to their criminality and alcohol and drug use, respectively.

## Discussion

The aim of the present study was to investigate the association between family psychosocial characteristics and the individual's development into criminal behaviour and mortality. Three research questions were included: first, how different family psychosocial characteristics in childhood may influence children's development into criminal behaviour and mortality; second, potential mediation by the individual's own alcohol and/or drug use and mental problems; and, third, possible differences in these patterns by gender.

### Influences on the outcome variable criminality

The different indicators of family psychosocial characteristics (father's criminality, father's alcohol abuse and parents' mental problems) were strongly associated with subsequent criminality in the male group in line with earlier research [8,49-51]. These associations might, apart from familial/genetic influences, also be interpreted as due to social modelling, as well as shared risk factors such as peer factors and individual features [52,53]. Among females, however, having a father abusing alcohol appeared to be more important for their criminal behaviour than the father's criminality, or either of the parents having mental problems. Worth noticing, these associations were also similar when only violent crimes were considered as the outcome (data not presented). These associations were largely unexplained by the occupational class during childhood. Interestingly, this is in line with earlier results from a Swedish study reporting the same influence of alcoholic fathers, comparing those from the highest- and lowest social class, on their children's development of juvenile delinquency [54]. Furthermore, in the present study, both for males and females, statistically significant links between individual problems, in terms of alcohol and/or drug use as well as mental problems, and criminality were found. This is of special interest, since these individual problems in the present study covered the age period of 13-19 years, and thus indicate that early-onset alcohol problems were closely connected to criminal behaviour and a family history of alcohol abuse [22]. Additionally, to some extent, these individual problems seemed to possibly mediate the association between family psychosocial characteristics and the individual's own subsequent criminality. It is noteworthy, however, that females' own alcohol and/or drug abuse overrode the effect of adverse family characteristics on criminality.

### Influences on the outcome variable mortality

The present results further suggested that deaths in the age range of 28 to 56 years are more common among males whose fathers abused alcohol as compared to their male counterparts. No corresponding associations seemed to apply for father's criminality or mental problems in the parents. The results can be compared with findings from another study [8] reporting parents' abuse of alcohol having consequences for their children (both males and females) during the age period of 15 to 27 years in terms of mortality and hospitalization due to violence. In the present study however, among females, parents' mental problems emerged as the most important of the family psychosocial characteristics investigated for the outcome of early death (this was the case regardless of whether these problems occurred in the mother or the father, data not presented). These are new findings and should be further studied. Childhood social class did not seem to confound this relationship to any large extent. With regard to individual problems, criminality (all crimes as well as violent crimes), alcohol and/or drug use, and, to a lesser extent, mental problems, contributed to parts of the explanation to the associations between family psychosocial characteristics and subsequent mortality. This was the case for both male and female subjects.

### Strengths and limitations

The prospective design of the Stockholm Birth Cohort database permitted the study of development into criminal behaviour and mortality. Moreover, the richness of the data made it possible to include a variety of indicators of family psychosocial characteristics and individuals problems. The large-scale data material also enabled us to perform gender-specific analysis in order to examine whether the patterns of association studied differed for males and females. There are nevertheless some issues that need to be recognised. Firstly, although the longitudinal data allowed for temporal ordering of the variables, no inference about causality can be made. The pathways leading to criminal behaviour and, not the least, to mortality are multifaceted and involve a complex interplay between family psychosocial characteristics and individual problems in the developmental context. Secondly, given the explorative approach of the present study, the investigated indicators were defined in terms of relatively broad categories. Future studies should aim at investigating these aspects in more detail, as well as including other sets of explanatory factors such as intelligence, other school-related indicators, and personality factors. Thirdly, the outcomes were captured through a rather lengthy follow-up (14 years for criminality and 28 years for mortality). The types of criminality and cause of death were likely to vary by age, which could make it more complicated to disentangle the contributions of the family-related- and individual risk indicators studied to subsequent criminal behaviour and mortality. Finally, although attrition was very low in the analysis, the study sample contains some individuals whose families were not resident in the actual Stockholm area before 1963, and some individuals who could have moved (in and) out of the area after 1963. The prevalence of family-related characteristics and individual problems could thus have been underestimated, which is likely also to cause an underestimation of the magnitude of the relationships obtained.

### Practical implications of the findings

The present study indicated that family psychosocial characteristics during childhood are associated with the child's subsequent criminal behaviour and mortality. To some extent, these influences appeared to operate through individual risk factors, such as alcohol and/or drug use and mental problems (for mortality also the risk factor of criminality). In order to give a more balanced picture, which reaches over and above the one that may be obtained by multivariate analysis, population attributable fractions were calculated. Results indicated that although the associations were strong, removing the exposure of father's alcohol abuse and criminality as well as parents' mental problems would not decrease the cases of criminality (a total decrease of 7.9% for males and 1.8% for females) or mortality (a total decrease of 1.2% for males as well as for females) to any large extent. Interestingly, however, having had no individual problems the incidence of criminality would have decreased greatly among males (a total decrease of 519 cases) or mortality (a total decrease of 53 cases), lesser so among the females (14 and 5 cases, respectively). Thus, the practical implications of the present study point to the importance of the individual's alcohol and/or drug use in reducing criminal behaviour, particularly among males. Moreover, any reduction in criminality would subsequently lower the mortality rates. This interpretation is however restricted to the framework of the current study. To eliminate the effect of adverse family psychosocial characteristics is still likely to be highly beneficial for other types of outcomes in the child, such as psychological well-being and school achievement, which in turn could have an impact on opportunities, achievements, and lifestyle in adulthood. Furthermore, there is still a substantial part of the development into criminal behaviour and subsequent mortality that remains unaccounted for by the risk factors included here. Thus, the findings of the present study should be considered in light of these arguments. It is also necessary to discuss whether our findings, as they are based on a Swedish cohort, are applicable to other settings. In comparison to many other countries, Sweden has a generous welfare system in part designed to compensate for unequal living conditions across social groups. This could possibly indicate that the safety net for individuals growing up in families with more adverse family psychosocial characteristics is more comprehensive compared to other cultural and societal settings.

## Conclusions

The findings of the present study suggested that family psychosocial characteristics were associated with the child's subsequent criminal behaviour and mortality. These connections partly seemed to be mediated by individual risk factors, especially by alcohol and/or drug use, somewhat differently operating in the male and female groups. The practical implications of the findings point to the importance of the individual's alcohol and/or drug use in reducing criminal behaviour, which according to the present findings would also lower the mortality rates.

## Competing interests

The authors declare that they have no competing interests.

## Authors' contributions

BaK, the scientific leader of the project, conducted the preliminary design, participated in presenting the results, provided the supervision and the interpretation of data, drafting of the manuscript, and gave the final design and shape of the manuscript. YA was responsible for the statistical analysis, participated in the study design, presentation of the results and discussion, and drafting of the manuscript. UB participated in the study design, background, discussion, and drafting of the manuscript. P-AR conducted the preliminary design and contributed to the drafting of the manuscript. All authors have read and approved the final manuscript.

## Authors' information

Britt af Klinteberg, PhD, Professor of Psychology, Stockholm University, and affiliated Professor with the Department of Women's and Children's Health, Karolinska Institutet, Stockholm; Ylva Almquist, PhD, post doctoral researcher at CHESS, Stockholm University/Karolinska Institutet, Stockholm; Ulla Beijer, PhD, researcher at the Department of Women's and Children's Health, Karolinska Institutet, Stocholm; Per-Anders Rydelius, MD, PhD, Professor of Pediatrics, Department of Women's and Children's Health, Karolinska Institutet, Stockholm.

## Pre-publication history

The pre-publication history for this paper can be accessed here:

http://www.biomedcentral.com/1471-2458/11/756/prepub
